# Comparative Prognostic Value of Systemic Inflammation-Based Biomarkers in Advanced Non-Small Cell Lung Cancer Treated with First-Line Immunotherapy

**DOI:** 10.3390/biomedicines14081825

**Published:** 2026-08-13

**Authors:** Şahin Bedir, Gülin Alkan Şen, Hamza Abbasov, Murad Guliyev, Erdem Sünger, Burçin Çakan Demirel, Nilay Şengül, Abdilkerim Oyman, Yakup Bozkaya, Ahmet Bilici, Hande Turna, Mustafa Özgüroğlu, Gökmen Umut Erdem

**Affiliations:** 1 Department of Medical Oncology, Gaziosmanpaşa Hastanesi, Istinye University, 34010 Istanbul, Turkey; droyman84@gmail.com (A.O.); dr_yakupbozkaya@hotmail.com (Y.B.); 2 Department of Medical Oncology, Cerrahpasa Medical Faculty Hospital, Istanbul University-Cerrahpasa, 34320 Istanbul, Turkey; gulinalkan@msn.com (G.A.Ş.); hamzaabbasov90@gmail.com (H.A.); drguliyev892@gmail.com (M.G.); hande.turna@gmail.com (H.T.); mozguroglu@gmail.com (M.Ö.); 3 Department of Medical Oncology, Istanbul Medipol Mega University Hospital, Istanbul Medipol University, 34214 Istanbul, Turkey; erdemsunger@gmail.com (E.S.); burcin.cakandemirel@gmail.com (B.Ç.D.); abilici@medipol.edu.tr (A.B.); 4 Department of Medical Oncology, Medicana Ataköy Hospital, 34158 Istanbul, Turkey; drnilaysengul@hotmail.com; 5 Department of Medical Oncology, Istanbul Başakşehir Çam ve Sakura Şehir Hastanesi, 34480 Istanbul, Turkey; gokmenumut@hotmail.com

**Keywords:** non-small cell lung cancer, immune checkpoint inhibitors, prognostic biomarkers

## Abstract

**Background:** We assessed the prognostic value of the pan-immune–inflammation value (PIV), systemic immune–inflammation index (SII), lung immune prognostic index (LIPI), and c-reactive protein–albumin–lymphocyte (CALLY) index in advanced non-small cell lung cancer (NSCLC) treated with first-line immune checkpoint inhibitor (ICI)-based therapy. **Methods:** This multicenter retrospective study included 161 patients who received ICI monotherapy, chemoimmunotherapy, or dual ICI therapy. The objective response rate (ORR), clinical benefit rate (CBR), progression-free survival (PFS), and overall survival (OS) were analyzed using Kaplan–Meier and Cox regression models. **Results:** A high CALLY index was associated with a significantly higher CBR (82.1% vs. 59.5%; *p* = 0.015), longer PFS (14.95 vs. 7.13 months; *p* = 0.002), and longer OS (25.79 vs. 13.24 months; *p* = 0.003). In the multivariable analyses, a high CALLY index remained independently associated with improved PFS (HR = 0.52, *p* = 0.006) and OS (HR = 0.53, *p* = 0.010), whereas a high SII was independently associated with poorer PFS (HR = 1.74, *p* = 0.021) and OS (HR = 1.90, *p* = 0.009). PIV and LIPI were not independently associated with survival outcomes. **Conclusions:** SII and the CALLY index emerged as independent prognostic biomarkers in advanced NSCLC receiving first-line immunotherapy-based treatment. The CALLY index showed the strongest and most consistent association with clinical benefit, PFS, and OS.

## 1. Introduction

Lung cancer remains the leading cause of cancer-related mortality worldwide, with non-small cell lung cancer (NSCLC) accounting for approximately 85% of all lung cancer diagnoses [1,2]. Immune checkpoint molecules expressed by tumor cells can suppress T-cell activation, thereby facilitating immune evasion and impairing the effectiveness of antitumor immune responses [3]. By disrupting the interaction between PD-1 and PD-L1, immune checkpoint inhibitors targeting this pathway have demonstrated substantial clinical efficacy and become an integral component of modern cancer therapy [4,5]. Nevertheless, identifying which patients are most likely to derive benefit from immunotherapy remains a major clinical challenge [6].

Tumor PD-L1 expression is an established biomarker used to predict treatment response in patients with advanced NSCLC [7,8]. However, the predictive utility of PD-L1 remains limited by intratumoral heterogeneity and discordant expression patterns between primary and metastatic tumors, as well as between biopsy and surgical resection specimens [9]. Similarly, although tumor mutational burden (TMB) has demonstrated predictive potential in several malignancies, the lack of standardized assessment methods and inconsistent clinical associations in NSCLC have limited its adoption as a reliable biomarker [10,11].

Inflammatory cytokines exert direct effects on tumor cells, promote epithelial–mesenchymal transition, and facilitate metastatic dissemination [12]. Inflammatory cells, including neutrophils, lymphocytes, monocytes, and platelets, may further contribute to tumor progression by promoting angiogenesis and cancer cell proliferation [13]. In this context, biomarkers such as the neutrophil-to-lymphocyte ratio (NLR) and platelet-to-lymphocyte ratio (PLR) have demonstrated prognostic value across various malignancies, including NSCLC; however, a single index may not fully capture the complexity of the systemic inflammatory response [14,15].

To improve prognostic risk stratification among patients receiving immune checkpoint inhibitor (ICI)-based therapy, there is a growing need for biomarkers that are accurate, readily accessible, and capable of reflecting the host immune milieu. The pan-immune–inflammation value (PIV), a composite index integrating neutrophil, lymphocyte, monocyte, and platelet counts, has emerged as a promising marker of systemic immune–inflammatory status and may provide valuable prognostic information in patients with cancer [16,17]. Similarly, the systemic immune–inflammation index (SII) has emerged as a clinically valuable prognostic biomarker, offering the advantages of cost-effectiveness and widespread availability in routine clinical practice [18,19]. Furthermore, the Lung Immune Prognostic Index (LIPI), a cost-effective biomarker derived from serum lactate dehydrogenase (LDH) levels and the derived neutrophil-to-lymphocyte ratio (dNLR), may serve as a surrogate measure of both tumor burden and deleterious systemic inflammation [20]. The C-reactive protein–albumin–lymphocyte (CALLY) index is another composite prognostic biomarker that integrates C-reactive protein (CRP), serum albumin, and peripheral lymphocyte counts. It reflects systemic inflammation, nutritional status, and host immune competence, thereby providing a comprehensive assessment of factors associated with cancer prognosis [21].

Previous studies and meta-analyses have generally evaluated PIV, SII, and LIPI separately and have confirmed their prognostic relevance across heterogeneous patient populations, treatment lines, therapeutic regimens, and cutoff definitions [17,20,22,23]. In addition, studies investigating the CALLY index in NSCLC have predominantly focused on patients undergoing surgery or thoracic radiotherapy, while evidence in metastatic disease treated with first-line immunotherapy remains limited [24,25,26,27]. Consequently, the relative clinical utility of these biomarkers when assessed concurrently within a uniform first-line immunotherapy setting remains unclear. The present study aimed to directly compare PIV, SII, LIPI, and the CALLY index within the same multicenter cohort of patients receiving ICI monotherapy, chemoimmunotherapy, or dual ICI therapy. Unlike previous reports focusing primarily on individual biomarkers and survival outcomes, we evaluated their associations with objective response rate, clinical benefit rate, progression-free survival, and overall survival using a consistent analytical framework. This head-to-head assessment represents the principal contribution of the current study and may help identify which readily available biomarker provides the most clinically relevant prognostic information in contemporary practice.

## 2. Materials and Methods

### 2.1. Patient Population

This retrospective cohort study included patients with advanced non-small cell lung cancer (NSCLC) who received programmed cell death protein 1 (PD-1) or programmed death-ligand 1 (PD-L1) inhibitors at Istinye University Gaziosmanpaşa Hospital, Istanbul University–Cerrahpaşa Cerrahpaşa Faculty of Medicine, and Medipol Mega University Hospital between January 2019 and January 2025.

The eligibility criteria for study inclusion were as follows: (1) histologically or cytologically confirmed advanced NSCLC; (2) receipt of PD-1/PD-L1 inhibitor therapy, either as monotherapy or in combination with other treatment modalities; (3) availability of complete clinical records, laboratory data, and radiological assessments throughout the treatment course; (4) the presence of at least one measurable lesion at baseline according to RECIST version 1.1.

The exclusion criteria were as follows: (1) the presence of a concurrent malignancy; (2) detection of actionable driver alterations, including mutations or rearrangements involving *EGFR, ALK, ROS1, RET,* or *MET*; (3) a documented history of autoimmune disease or immunodeficiency.

### 2.2. Data Collection and Definitions

Demographic and clinical data were retrospectively extracted from electronic medical records, including age, sex, smoking history, Eastern Cooperative Oncology Group performance status (ECOG-PS), histological subtype, clinical stage, the presence of brain, liver, bone, pleural, or adrenal metastases, PD-L1 tumor proportion score (TPS), as well as the chemotherapy and immunotherapy regimens administered.

To evaluate inflammatory biomarkers, complete blood count (CBC) parameters obtained from peripheral blood samples collected within 7 days prior to the initiation of PD-1/PD-L1 inhibitor therapy were analyzed.

Tumor PD-L1 expression was assessed in pretreatment tumor specimens using the PD-L1 22C3 pharmDx immunohistochemistry assay (Agilent Technologies, Inc., Santa Clara, CA, USA). PD-L1 TPS was descriptively categorized as negative (<1%), low expression (1–49%), and high expression (≥50%). For Cox regression analyses, PD-L1 TPS was dichotomized as <50% versus ≥50%, because the ≥50% threshold is clinically relevant for first-line treatment selection and the small number of patients with PD-L1 TPS < 1% precluded stable three-category estimates.

The ICI monotherapy regimens included pembrolizumab and atezolizumab. Chemoimmunotherapy consisted primarily of pembrolizumab combined with a platinum agent (carboplatin or cisplatin) and pemetrexed for patients with nonsquamous histology, or pembrolizumab combined with carboplatin and paclitaxel for patients with squamous histology. Pemetrexed and pembrolizumab or pembrolizumab alone were continued as maintenance therapy, as appropriate. Dual ICI-based treatment consisted of nivolumab plus ipilimumab, with or without a limited course of platinum-based chemotherapy. Treatment selection was determined by the treating physician according to histology, PD-L1 expression, disease characteristics, organ function, and patient fitness.

PIV was calculated using the following formula: (neutrophil count × platelet count × monocyte count)/lymphocyte count. SII was calculated as (neutrophil count × platelet count)/lymphocyte count.

LIPI was determined based on two pretreatment risk factors: a dNLR (neutrophil count/[white blood cell count − neutrophil count]) > 3 and a LDH level above the upper limit of normal. Patients were subsequently categorized into three prognostic groups according to the number of risk factors present: good (0 risk factors), intermediate (1 risk factor), and poor (2 risk factors).

The CALLY index was calculated using the formula: serum albumin concentration (g/dL) × absolute lymphocyte count (/μL)/[CRP (mg/dL) × 10^4^].

### 2.3. Treatment Response and Survival Outcomes

Overall survival (OS) was defined as the interval between the initiation of immunotherapy and death from any cause. Progression-free survival (PFS) was defined as the time from treatment initiation to documented disease progression or death from any cause, whichever occurred first.

All patients had at least one measurable lesion at baseline, and radiological treatment response was evaluated according to RECIST version 1.1. Radiological response assessments were generally performed at baseline and every 12 weeks during treatment, according to routine clinical practice at the participating centers. Additional imaging was performed earlier when clinically indicated because of suspected disease progression. Responses were categorized as complete response (CR), partial response (PR), stable disease (SD), or progressive disease (PD). The objective response rate (ORR) was defined as the proportion of patients achieving either CR or PR, whereas the clinical benefit rate (CBR) was defined as the proportion achieving CR, PR, or SD.

### 2.4. Statistical Analysis

Baseline characteristics were compared between subgroups using the chi-square test or Fisher’s exact test for categorical variables and Student’s *t*-test for continuous variables. Optimal cutoff values for PIV, SII, and the CALLY index were determined using receiver operating characteristic (ROC) curve analysis, with vital status at the last follow-up (alive versus deceased) as the binary outcome. The thresholds maximizing the Youden index (sensitivity + specificity − 1) were selected. The resulting cutoff values were 947.1 for PIV, 1212.7 for SII, and 0.0275 for the CALLY index. Patients were subsequently categorized into high and low groups according to these cohort-derived thresholds. Previously published cutoff values were considered but were not adopted because of substantial heterogeneity in patient populations, treatment settings, study endpoints, laboratory units, and biomarker calculation methods.

Survival outcomes were estimated using the Kaplan–Meier method and compared between groups using the log-rank test. Median follow-up was calculated using the reverse Kaplan–Meier method. Cox proportional hazards regression models were used to identify independent prognostic factors associated with PFS and OS. Variables with a *p* value < 0.10 in the univariable analyses were entered simultaneously into the multivariable models using the enter method; no backward or forward selection procedure was applied. Multicollinearity among the included variables was assessed using variance inflation factors and tolerance values. Exploratory Spearman rank correlation analysis was performed to assess the relationships among continuous PIV, SII, and CALLY values and the ordinal LIPI score. The proportional hazards assumption was evaluated by testing interactions between the covariates and log-transformed survival time. Model calibration and discrimination were not assessed because the analyses were intended to identify independent prognostic associations rather than to develop an individualized risk prediction model.

A two-sided *p* value of <0.05 was considered statistically significant. All statistical analyses were performed using SPSS for Windows, version 26.0 (IBM Corp., Chicago, IL, USA).

### 2.5. Ethics Approval

This study was conducted in accordance with the principles of the Declaration of Helsinki and was approved by the Ethics Committee of Yeni Yüzyıl University on 3 February 2026 (Approval No. 2026/02-1842). Given the retrospective nature of the study and the use of anonymized patient data, the requirement for informed consent was waived by the Ethics Committee.

## 3. Results

### 3.1. Patient Characteristics

**Figure 1:**
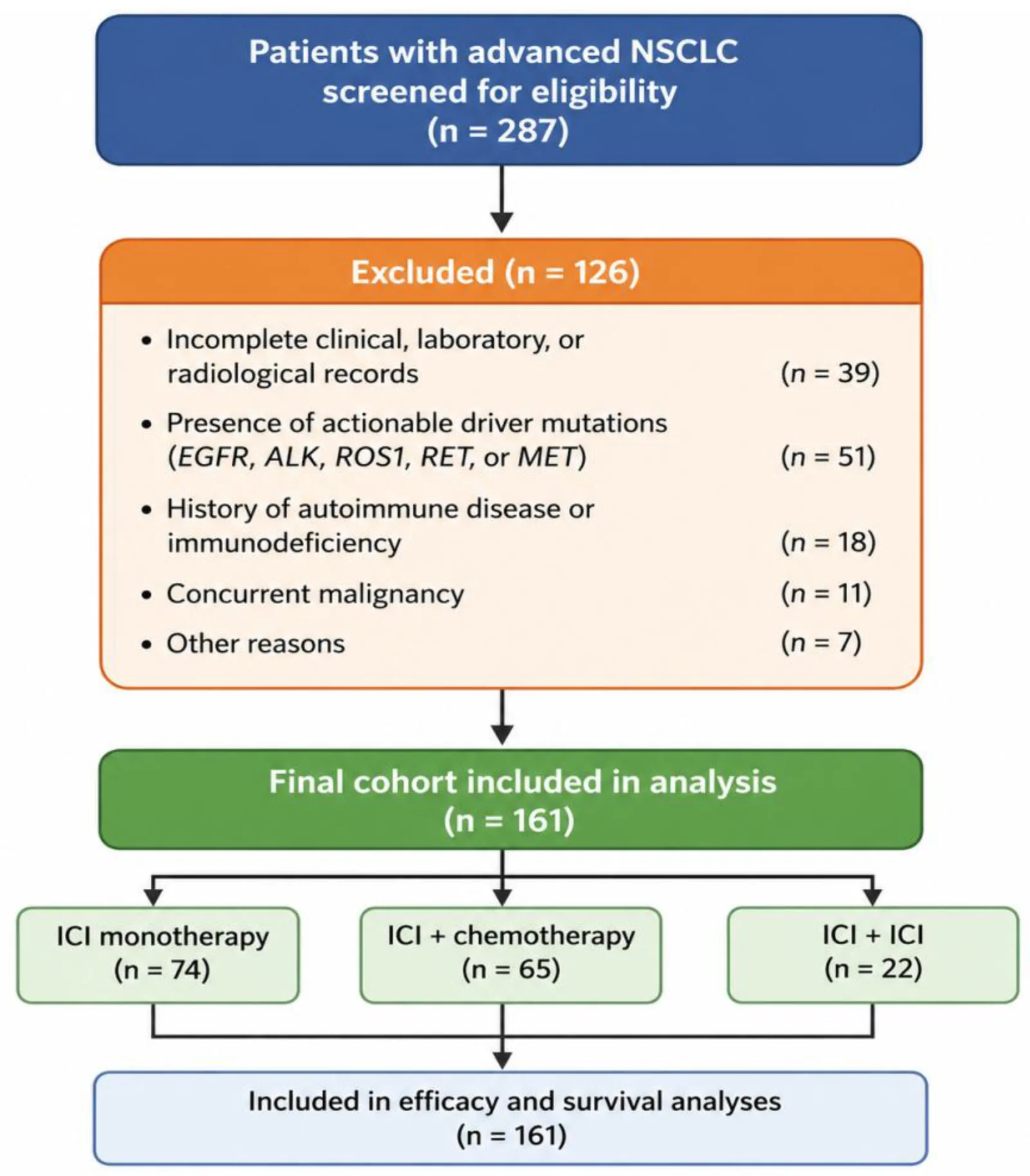
Patient selection flow diagram. Of 287 patients with advanced non-small cell lung cancer screened for eligibility, 126 were excluded and 161 were included in the final analysis. Patients received immune checkpoint inhibitor monotherapy (*n* = 74), chemoimmunotherapy (*n* = 65), or dual immune checkpoint blockade (*n* = 22). NSCLC, non-small cell lung cancer; ICI, immune checkpoint inhibitor.

We retrospectively analyzed the clinicopathological characteristics of 161 patients with advanced NSCLC who received either ICI monotherapy or ICI-based combination therapy with chemotherapy as first-line treatment. As shown in Figure 1, 287 patients were screened, of whom 126 were excluded according to predefined criteria. The final cohort comprised 74 patients treated with ICI monotherapy, 65 with ICI plus chemotherapy, and 22 with dual ICI therapy.

The median age of the study population was 63 years (range, 34–82), and 71 patients (44.1%) were aged ≥65 years. Most patients were male (*n* = 129, 80.1%). An Eastern Cooperative Oncology Group performance status (ECOG-PS) of 0–1 was observed in 115 patients (71.4%), while a history of smoking was documented in 145 patients (89.8%).

Histopathological evaluation revealed non-squamous histology in 115 patients (71.5%), squamous cell carcinoma in 35 (21.7%), and non-specific carcinoma in 11 (6.9%). De novo metastatic disease was present in 129 patients (80.1%), whereas 32 patients (19.9%) had recurrent disease. Regarding metastatic distribution, visceral metastases were identified in 114 patients (70.8%), while 47 patients (29.2%) had non-visceral metastatic involvement.

PD-L1 expression was classified as high (TPS ≥ 50%) in 71 patients (44.1%), low (TPS 1–49%) in 75 patients (46.6%), and negative (TPS < 1%) in 15 patients (9.3%). According to the PIV classification, 79 patients (49.1%) were assigned to the high-PIV (H-PIV) group and 82 patients (50.9%) to the low-PIV (L-PIV) group. Based on SII, 77 patients (47.8%) were categorized as high-SII (H-SII) and 84 (52.2%) as low-SII (L-SII). According to the LIPI classification, 54 patients (33.5%) were classified as having good LIPI, 86 (53.4%) with intermediate LIPI, and 21 (13.1%) with poor LIPI. For the CALLY index, 90 patients (55.9%) were categorized as high-CALLY (H-CALLY) and 71 (44.1%) as low-CALLY (L-CALLY).

Regarding treatment, 65 patients (40.4%) received chemoimmunotherapy, 74 (46.0%) received immune checkpoint inhibitor monotherapy, and 22 (13.6%) received dual immune checkpoint blockade.

### 3.2. Correlations Among Inflammation-Based Biomarkers

Exploratory Spearman correlation analysis demonstrated a very strong positive correlation between SII and PIV (ρ = 0.897, *p* < 0.001). CALLY was moderately and negatively correlated with SII (ρ = −0.524, *p* < 0.001) and PIV (ρ = −0.434, *p* < 0.001). LIPI showed a moderate positive correlation with SII (ρ = 0.345, *p* < 0.001), a weak positive correlation with PIV (ρ = 0.260, *p* = 0.003), and a moderate negative correlation with CALLY (ρ = −0.382, *p* < 0.001). These findings indicate substantial biological and mathematical overlap between PIV and SII, whereas CALLY and LIPI may capture partially distinct inflammatory and immunonutritional information.

### 3.3. Response Outcomes

All patients received first-line (ICI)-based treatment for advanced NSCLC, including ICI monotherapy, ICI-based chemoimmunotherapy, or dual immune checkpoint blockade. Among the 161 evaluable patients, no significant association was observed between PIV status and ORR. The ORR was 44.0% in the high-PIV (H-PIV) group and 42.9% in the low-PIV (L-PIV) group (OR = 1.05, 95% CI: 0.55–1.99, *p* = 0.887). Similarly, ORR did not differ significantly between the high-SII (H-SII) and low-SII (L-SII) groups, with response rates of 39.7% and 47.5%, respectively (OR = 0.73, 95% CI: 0.38–1.38, *p* = 0.334).

According to LIPI classification, ORRs were 46.5%, 45.6%, and 47.1% in the good, intermediate, and poor LIPI groups, respectively. No significant differences in ORR were observed across LIPI categories. Compared with the poor-LIPI group, neither the good-LIPI group (OR = 0.98, 95% CI: 0.32–3.01, *p* = 0.969) nor the intermediate-LIPI group (OR = 0.94, 95% CI: 0.33–2.74, *p* = 0.913) demonstrated a statistically significant association with treatment response.

The ORR was 42.9% in the high-CALLY (H-CALLY) group and 26.2% in the low-CALLY (L-CALLY) group. Although a numerically higher response rate was observed among patients with a high CALLY index, the difference did not reach statistical significance (OR = 2.11, 95% CI: 0.89–5.03, *p* = 0.091).

CBRs were 73.3% and 70.1% in the H-PIV and L-PIV groups, respectively, with no significant association between PIV status and clinical benefit (OR = 1.17, 95% CI: 0.58–2.38, *p* = 0.661). Likewise, CBRs were 71.2% in the H-SII group and 72.5% in the L-SII group, and no statistically significant relationship was identified between SII status and clinical benefit (OR = 0.94, 95% CI: 0.46–1.90, *p* = 0.862).

CBRs according to LIPI classification were 79.1%, 70.6%, and 82.3% in the good-, intermediate-, and poor-LIPI groups, respectively. No significant association was found between LIPI category and clinical benefit. Relative to the poor-LIPI group, neither the good-LIPI group (OR = 0.81, 95% CI: 0.19–3.44, *p* = 0.775) nor the intermediate-LIPI group (OR = 0.51, 95% CI: 0.13–1.99, *p* = 0.335) showed a significant correlation with CBR.

In contrast, patients with a high CALLY index achieved a significantly higher clinical benefit rate than those with a low CALLY index (82.1% vs. 59.5%; OR = 3.13, 95% CI: 1.25–7.85, *p* = 0.015).

### 3.4. Progression-Free Survival

The median follow-up duration for the entire cohort was 16.98 months (range, 1.84–112.85). During the follow-up period, disease progression was documented in 112 patients (69.6%). The median PFS for the overall study population was 9.95 months (95% CI: 6.99–12.91).

According to PIV status, the median PFS was 9.10 months (95% CI: 7.14–11.06) in the high-PIV (H-PIV) group and 12.19 months (95% CI: 8.23–16.15) in the low-PIV (L-PIV) group. However, Kaplan–Meier analysis did not demonstrate a statistically significant association between PIV category and PFS (log-rank *p* = 0.871).

Similarly, the median PFS was 8.41 months (95% CI: 6.63–10.19) in the high-SII (H-SII) group and 14.95 months (95% CI: 8.80–21.09) in the low-SII (L-SII) group. No significant difference in PFS was observed between SII subgroups on Kaplan–Meier analysis (log-rank *p* = 0.053).

When stratified according to LIPI, median PFS was 10.31 months (95% CI: 4.70–15.92) in the good-LIPI group, 9.79 months (95% CI: 5.14–14.43) in the intermediate-LIPI group, and 9.10 months (95% CI: 5.43–12.76) in the poor-LIPI group. Kaplan–Meier analysis revealed no significant association between LIPI classification and PFS (log-rank *p* = 0.643).

**Figure 2:**
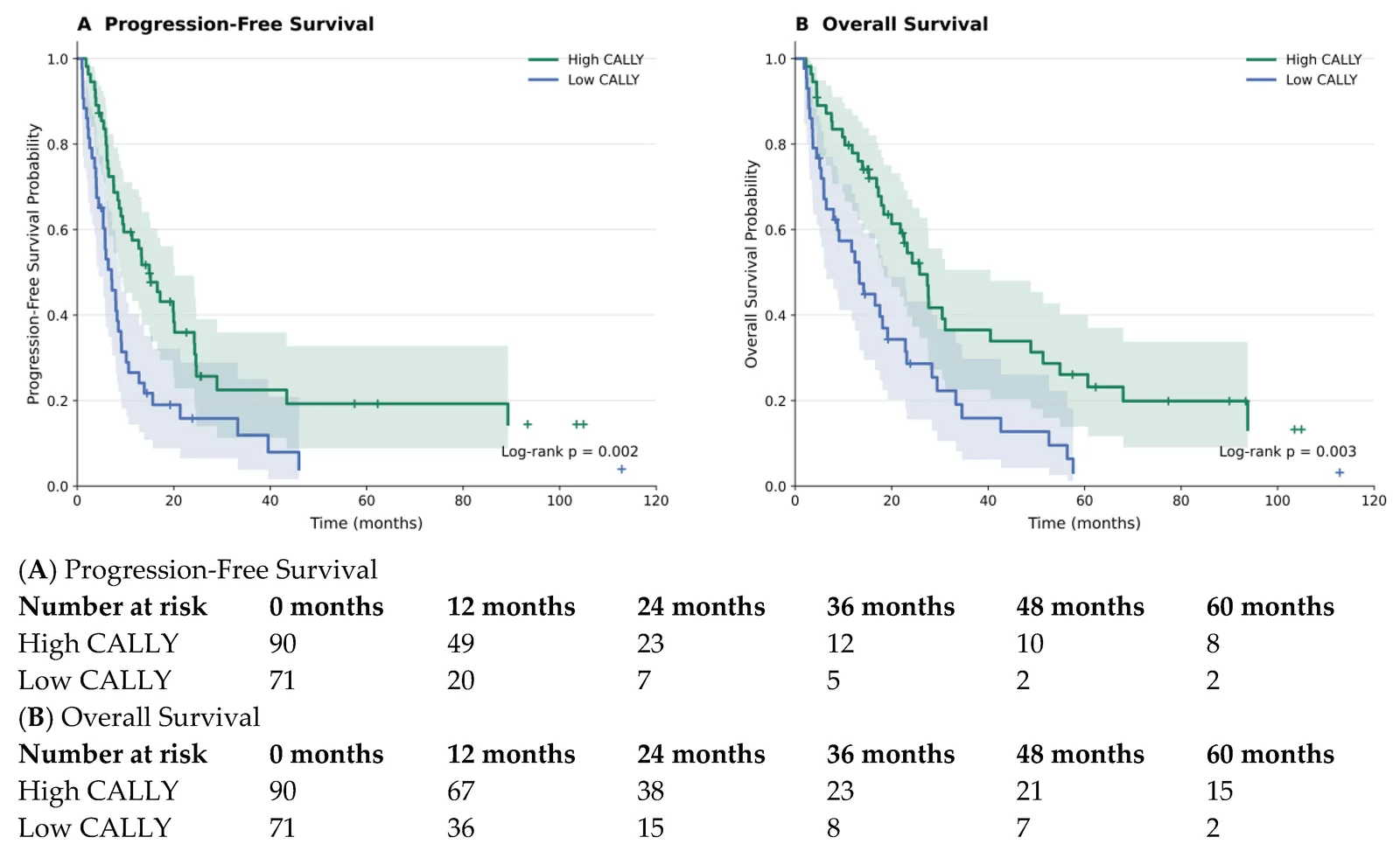
Kaplan–Meier survival curves according to the C-reactive protein–albumin–lymphocyte (CALLY) index. (**A**) Progression-free survival was significantly longer in the high-CALLY group than in the low-CALLY group (median, 14.95 vs. 7.13 months; log-rank *p* = 0.002). (**B**) Overall survival was significantly longer in the high-CALLY group than in the low-CALLY group (median, 25.79 vs. 13.24 months; log-rank *p* = 0.003). Numbers at risk are presented below each plot. Shaded areas represent 95% confidence intervals, and colored plus signs (+ and ++) indicate censored observations. CALLY, C-reactive protein–albumin–lymphocyte index.

In contrast, patients in the high-CALLY (H-CALLY) group experienced significantly longer PFS compared with those in the low-CALLY (L-CALLY) group. The median PFS was 14.95 months (95% CI: 10.16–19.73) in the H-CALLY group and 7.13 months (95% CI: 4.46–9.80) in the L-CALLY group. Kaplan–Meier analysis confirmed a statistically significant improvement in PFS among patients with high CALLY index (log-rank *p* = 0.002) (Figure 2A).

**Figure 3:**
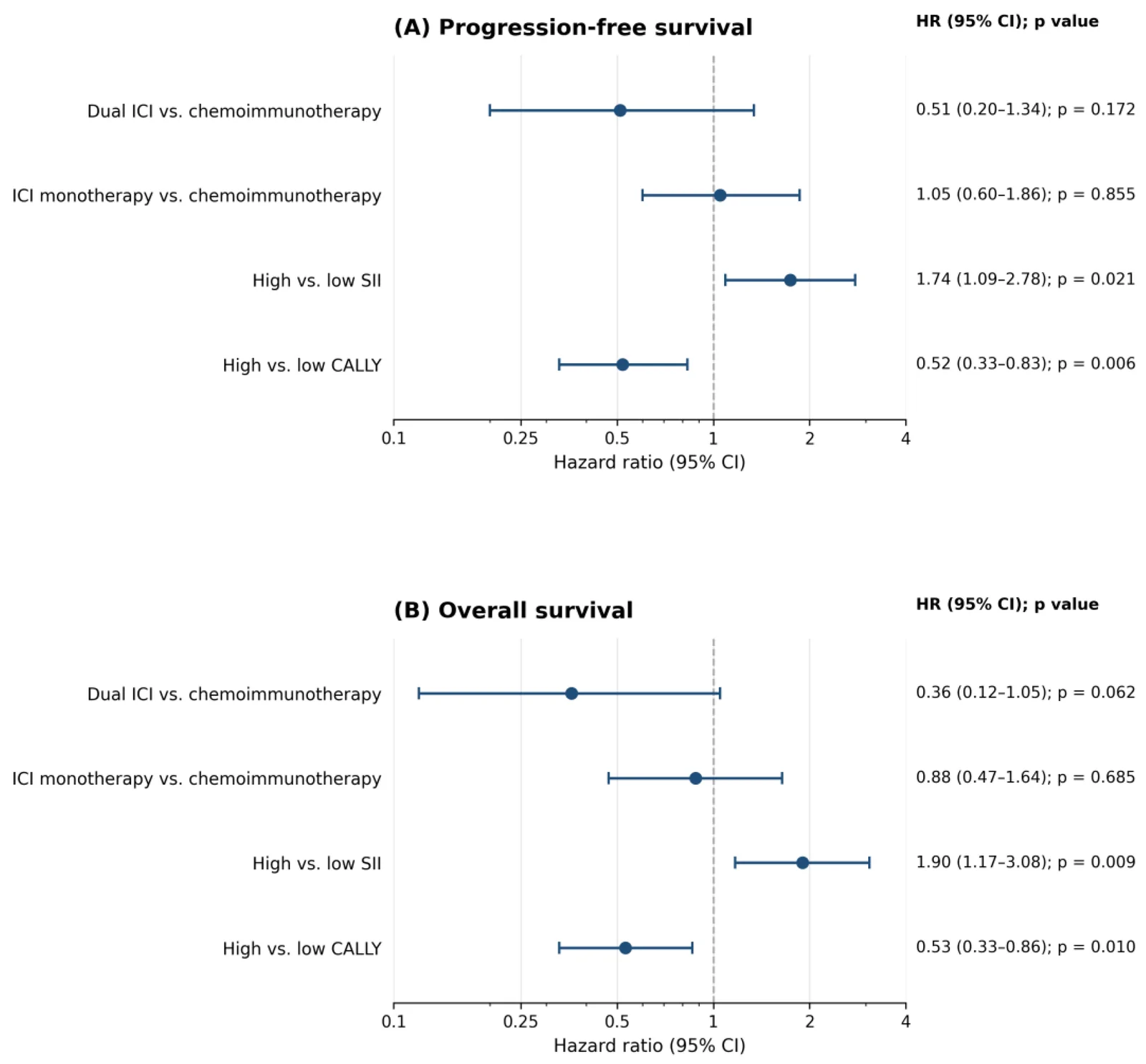
Forest plots of the multivariable Cox regression analyses for (**A**) progression-free survival and (**B**) overall survival. HR, hazard ratio; CI, confidence interval; ICI, immune checkpoint inhibitor; SII, systemic immune–inflammation index; CALLY, C-reactive protein–albumin–lymphocyte index.

In univariable Cox regression analysis, a high CALLY index was significantly associated with prolonged progression-free survival. Although high SII showed only a borderline association with PFS in the univariable analysis, both variables remained independently associated with PFS in the multivariable model including treatment type, SII, and CALLY. High CALLY status was independently associated with improved PFS (HR = 0.52, 95% CI: 0.33–0.83, *p* = 0.006), whereas high SII was independently associated with an increased risk of progression (HR = 1.74, 95% CI: 1.09–2.78, *p* = 0.021) (Table 1). The results of the multivariable Cox regression analysis for PFS are visually summarized in Figure 3A.

### 3.5. Overall Survival

During the follow-up period, 99 patients (61.5%) died. The median OS for the entire cohort was 23.13 months (95% CI: 17.24–29.02).

According to PIV status, the median OS was 18.10 months (95% CI: 9.33–26.87) in the high-PIV (H-PIV) group and 27.66 months (95% CI: 19.53–35.80) in the low-PIV (L-PIV) group. However, Kaplan–Meier analysis did not demonstrate a statistically significant association between PIV category and OS (log-rank *p* = 0.163).

The median OS was 17.54 months (95% CI: 12.41–22.68) in the high-SII (H-SII) group and 28.35 months (95% CI: 24.02–32.68) in the low-SII (L-SII) group. Kaplan–Meier analysis revealed a significantly longer OS among patients in the L-SII group (log-rank *p* = 0.028).

When stratified according to LIPI classification, the median OS was 27.60 months (95% CI: 11.32–43.87) in the good-LIPI group, 27.40 months (95% CI: 21.03–33.77) in the intermediate-LIPI group, and 16.56 months (95% CI: 5.10–28.01) in the poor-LIPI group. Nevertheless, no statistically significant differences in OS were observed among the LIPI subgroups (log-rank *p* = 0.265).

In contrast, patients with high CALLY index experienced markedly improved survival outcomes compared with those with low CALLY index. The median OS was 25.79 months (95% CI: 19.99–31.58) in the high-CALLY (H-CALLY) group and 13.24 months (95% CI: 7.10–19.37) in the low-CALLY (L-CALLY) group. Kaplan–Meier analysis confirmed that high CALLY status was significantly associated with prolonged OS (log-rank *p* = 0.003) (Figure 2B).

In univariable Cox regression analysis, high SII and low CALLY index were significantly associated with poorer overall survival. In the multivariable model, both biomarkers retained their independent prognostic significance. High SII was independently associated with an increased risk of death (HR = 1.90, 95% CI: 1.17–3.08, *p* = 0.009), whereas high CALLY status was independently associated with improved overall survival (HR = 0.53, 95% CI: 0.33–0.86, *p* = 0.010) (Table 2). The results of the multivariable Cox regression analysis for OS are visually summarized in Figure 3B.

## 4. Discussion

In the present study, we evaluated the prognostic significance of inflammation-based biomarkers in patients with metastatic NSCLC receiving first-line immunotherapy-based treatment. Both SII and the CALLY index were independently associated with clinical outcomes. A high SII was associated with shorter PFS and OS, whereas a high CALLY index was associated with prolonged PFS and OS. In addition, patients with a high CALLY index achieved a significantly higher clinical benefit rate. These findings underscore the importance of systemic inflammation and host immunonutritional status in determining clinical outcomes among patients receiving immunotherapy-based treatment.

Among the evaluated biomarkers, SII emerged as a significant independent prognostic factor for both PFS and OS. SII reflects the balance between protumor inflammatory processes and host antitumor immune activity by integrating neutrophil, platelet, and lymphocyte counts into a single index. Elevated neutrophil and platelet levels have been shown to promote tumor growth, angiogenesis, immune evasion, and metastatic dissemination, whereas lymphocytes play a pivotal role in antitumor immune surveillance and immune checkpoint inhibitor efficacy [12,13]. Consequently, a high SII may indicate an immunosuppressive tumor microenvironment associated with unfavorable clinical outcomes. Our findings are consistent with previous reports demonstrating the prognostic value of SII in patients with advanced NSCLC receiving immunotherapy-based treatment [28,29,30]. Although some studies have reported heterogeneous results across different treatment settings and patient populations, the overall body of evidence supports the role of SII as a robust marker of systemic inflammation and adverse prognosis in advanced NSCLC.

In contrast, neither PIV nor LIPI was significantly associated with survival in our cohort. This differs from recent evidence supporting the prognostic value of both markers. Chen et al. reported that lower baseline and early on-treatment PIVs were associated with longer PFS and OS in patients with advanced NSCLC receiving immunotherapy [31]. Similarly, a recent meta-analysis found that an elevated LIPI was associated with poorer OS and PFS in patients with NSCLC treated with ICIs [22]. However, differences in patient selection, treatment regimens, biomarker assessment timing, cutoff definitions, and the distribution of LIPI risk groups may explain the discrepancy with our results. The inclusion of ICI monotherapy, chemoimmunotherapy, and dual ICI therapy may also have attenuated biomarker-specific associations. Therefore, our findings suggest that the prognostic performance of PIV and LIPI may be context dependent and requires prospective validation using standardized thresholds.

The CALLY index demonstrated the strongest and most consistent prognostic performance among the evaluated biomarkers. Patients with a high CALLY index experienced significantly prolonged PFS and OS and achieved a higher clinical benefit rate. Importantly, the prognostic significance of CALLY remained independent of treatment type and SII in multivariable analyses, suggesting that this index captures complementary biological information beyond systemic inflammation alone. The prognostic relevance of the CALLY index in NSCLC has attracted increasing attention in recent years. Liu et al. demonstrated that the CALLY index independently associated overall survival in patients with NSCLC [24]. More recently, Yoneyama et al. and Mizota et al. reported significantly improved survival outcomes among patients with elevated CALLY index following surgical treatment for NSCLC [25,26]. Furthermore, Yin et al. showed that post-treatment CALLY values were associated with long-term outcomes in patients with locally advanced NSCLC treated with thoracic radiotherapy [27]. Our findings extend the current literature by demonstrating that CALLY is associated not only with survival outcomes but also with clinical benefit in patients receiving first-line immunotherapy-based treatment.

The CALLY index should primarily be interpreted as an integrative marker of the patient’s general inflammatory, nutritional, and immune status. Elevated CRP reflects systemic inflammation, whereas hypoalbuminemia may indicate malnutrition, systemic catabolism, cachexia, or reduced physiological reserve. Peripheral lymphocyte counts provide additional information regarding host immune competence. Accordingly, a high CALLY index may identify patients with lower inflammatory burden, preserved nutritional status, lower disease burden, and better general condition. These characteristics are associated with favorable outcomes across cancer treatments and are not necessarily specific to immune checkpoint inhibitor efficacy. Therefore, the association between CALLY and clinical outcomes in this cohort should be interpreted as prognostic rather than as evidence of an immunotherapy-specific biological effect.

The available evidence regarding the clinical utility of the CALLY index in metastatic NSCLC remains limited, particularly among patients receiving first-line immunotherapy-based treatment. Most previous studies have primarily focused on survival outcomes, whereas relatively few investigations have simultaneously evaluated treatment response, clinical benefit, progression-free survival, and overall survival. In this context, our findings suggest that the CALLY index may represent a practical and inexpensive integrative prognostic marker. However, it should not currently be considered an established biomarker for treatment selection, and its clinical utility requires prospective external validation.

The principal contribution of this study lies not in evaluating a single inflammatory biomarker in isolation, but in directly comparing four biologically distinct indices within the same contemporary first-line immunotherapy cohort. Previous meta-analyses have established the prognostic relevance of individual markers such as SII and PIV; however, comparisons across separate studies are limited by differences in patient selection, treatment line, therapeutic regimen, cutoff determination, and statistical adjustment [22,23]. By calculating PIV, SII, LIPI, and CALLY from the same pretreatment period and evaluating them against multiple clinical endpoints within a consistent analytical framework, our study reduces this cross-study heterogeneity. The finding that SII and CALLY, but not PIV or LIPI, retained independent prognostic significance suggests that these indices provide different—and potentially complementary—information rather than being interchangeable measures of systemic inflammation.

The correlation analysis demonstrated that these indices reflect overlapping but not identical biological processes. The very strong correlation between PIV and SII is expected because both indices incorporate neutrophil, platelet, and lymphocyte counts. In contrast, the moderate inverse correlations of CALLY with PIV, SII, and LIPI suggest that CALLY may capture partially distinct information through the inclusion of CRP and albumin. Nevertheless, these correlations do not establish the incremental prognostic value of CALLY beyond its individual components. Although SII and CALLY both retained significance in the multivariable survival models, formal model-comparison analyses were not performed; therefore, claims of biomarker superiority should be interpreted cautiously.

Treatment selection in this real-world cohort was influenced by multiple clinical factors, including PD-L1 expression, histology, disease burden, symptoms, organ function, and patient fitness. Therefore, the treatment groups represent clinically distinct populations, and adjustment for treatment category cannot completely eliminate confounding by indication. Further stratification by treatment category and biomarker status would have generated small subgroups and unstable effect estimates, particularly in the dual ICI group. Accordingly, the present findings should be interpreted as overall prognostic associations within a heterogeneous cohort receiving first-line ICI-based therapy rather than as evidence of uniform biomarker performance across individual treatment strategies.

From a clinical perspective, SII and CALLY can be calculated from routinely available pretreatment blood tests without additional cost. In the overall cohort, these biomarkers may complement established clinical factors by identifying patients with a higher-risk prognosis who may benefit from closer clinical and radiological monitoring; however, their utility within individual treatment categories remains uncertain. They should not currently be used alone to select or modify immunotherapy, as the cutoff values require external validation. Their potential role is therefore complementary risk stratification rather than treatment selection.

### Strengths and Limitations

This study has several strengths, including its multicenter design, the concurrent evaluation of four inflammation-based biomarkers, and the assessment of multiple clinical endpoints. However, its retrospective design entails risks of selection bias and residual confounding. The inclusion of ICI monotherapy, chemoimmunotherapy, and dual ICI therapy introduced treatment heterogeneity and confounding by indication that could not be fully eliminated through multivariable adjustment. Small subgroup sizes, particularly in the dual ICI group (*n* = 22), precluded reliable treatment-specific and biomarker-by-treatment interaction analyses. Therefore, the findings should not be interpreted as demonstrating uniform biomarker performance across treatment regimens or immunotherapy-specific predictive utility. Although PD-L1 TPS was reported as <1%, 1–49%, and ≥50%, it was dichotomized as <50% versus ≥50% in the Cox models, potentially obscuring differences between PD-L1-negative and low-positive disease. Furthermore, the multivariable Cox regression models were primarily constructed using variables selected according to a univariable *p* value < 0.10. Consequently, clinically relevant factors that did not meet this predefined threshold, including ECOG performance status, PD-L1 expression, histology, and measures of metastatic burden, were not necessarily retained in the final models. Residual confounding by these factors cannot be completely excluded; therefore, the independent prognostic associations observed for SII and the CALLY index should be interpreted with appropriate caution. CALLY may also reflect general inflammation, nutritional status, tumor burden, and frailty independently of treatment. Intercenter variability and insufficient patient-level imaging data limited standardized assessments and precluded reliable waterfall and swimmer plots. Biomarkers were assessed only at baseline, and the internally optimized cutoffs may be affected by overfitting and optimism bias. The conventional ROC analysis did not account for follow-up duration or censoring, and sensitivity analyses using alternative thresholds or continuous-variable modeling were not performed. The CALLY cutoff of 0.0275 should therefore be considered cohort-specific and exploratory. Finally, the absence of external validation limits generalizability. Larger prospective studies using standardized serial assessments, predefined thresholds, clinically adjusted models, and independent validation cohorts are warranted.

## 5. Conclusions

In conclusion, our findings indicate that inflammation-based biomarkers may provide clinically meaningful prognostic information in patients with advanced NSCLC receiving first-line immunotherapy-based treatment. In the overall cohort, SII and the CALLY index were independently associated with survival outcomes, whereas PIV and LIPI were not; however, the prognostic performance of these biomarkers within individual first-line treatment categories remains uncertain. Notably, a high CALLY index was associated with greater clinical benefit and prolonged progression-free and overall survival, whereas an elevated SII was associated with adverse outcomes. These findings support CALLY as a promising integrative prognostic marker of host inflammatory and nutritional status rather than an immunotherapy-specific predictive biomarker. CALLY should not currently be used for treatment selection, and its clinical utility requires prospective external validation using predefined thresholds. Future studies should investigate the integration of inflammatory biomarkers with molecular tumor characteristics and circulating tumor DNA. Artificial intelligence-based models combining these biomarkers with molecular and clinical data may further improve individualized prognostic stratification. Nevertheless, prospective multicenter studies with larger patient populations are warranted to validate these findings and to further define the role of these biomarkers alongside established clinicopathological and molecular biomarkers, including PD-L1 expression.

## Figures and Tables

**Table 1:** Prognostic factors associated with progression-free survival in univariable and multivariable Cox regression analyses.

		Univariable Analysis		Multivariable Analysis	
Variables	*n*	HR (95% CI)	*p* Value	HR (95% CI)	*p* Value
Age (≥65 vs. <65 years)	71/90	1.12 (0.77–1.63)	0.554		
Sex (male vs. female)	129/32	1.11 (0.69–1.79)	0.670		
ECOG PS (0–1 vs. 2)	115/46	0.82 (0.62–1.09)	0.157		
Histology					
Nonsquamous vs. squamous	115/35	1.47 (0.92–2.37)	0.107		
Disease status					
Recurrent vs. de novo metastatic	32/129	0.70 (0.43–1.13)	0.145		
Metastatic site					
Nonvisceral vs. visceral	47/114	1.02 (0.67–1.53)	0.940		
Liver metastasis (no vs. yes)	143/18	1.42 (0.82–2.45)	0.209		
Brain metastasis (no vs. yes)	127/34	1.02 (0.64–1.62)	0.950		
Bone metastasis (no vs. yes)	88/73	1.07 (0.74–1.56)	0.710		
PD-L1 TPS (≥50% vs. <50%)	71/90	0.77 (0.51–1.16)	0.213		
Treatment type			0.067		0.255
Dual ICI vs. chemoimmunotherapy	22/65	0.52 (0.26–1.04)	0.066	0.51 (0.20–1.34)	0.172
ICI monotherapy vs. chemoimmunotherapy	74/65	1.15 (0.77–1.73)	0.487	1.05 (0.60–1.86)	0.855
PIV (high vs. low)	79/82	1.03 (0.71–1.51)	0.871		
SII (high vs. low)	77/84	1.45 (0.99–2.13)	0.054	1.74 (1.09–2.78)	0.021
LIPI group			0.645		
Intermediate vs. poor	86/21	0.88 (0.48–1.63)	0.685		
Good vs. poor	54/21	0.75 (0.39–1.44)	0.385		
CALLY (high vs. low)	90/71	0.51 (0.32–0.79)	0.003	0.52 (0.33–0.83)	0.006

Abbreviations: HR, hazard ratio; CI, confidence interval; ECOG PS, Eastern Cooperative Oncology Group performance status; PD-L1 TPS, programmed death-ligand 1 tumor proportion score; ICI, immune checkpoint inhibitor; PIV, pan-immune–inflammation value; SII, systemic immune–inflammation index; LIPI, Lung Immune Prognostic Index; CALLY, C-reactive protein–albumin–lymphocyte index. HRs are presented with 95% CIs. Sample sizes for binary comparisons are reported in the same order as the categories listed in the Variables column. Variables with *p* < 0.10 in the univariable analysis were entered into the multivariable model.

**Table 2:** Prognostic factors associated with overall survival in univariable and multivariable Cox regression analyses.

		Univariable Analysis		Multivariable Analysis	
Variables	*n*	HR (95% CI)	*p* Value	HR (95% CI)	*p* Value
Age (≥65 vs. <65 years)	71/90	1.28 (0.86–1.91)	0.224		
Sex (male vs. female)	129/32	1.03 (0.62–1.70)	0.913		
ECOG PS (0–1 vs. 2)	115/46	0.87 (0.65–1.79)	0.370		
Histology					
Nonsquamous vs. squamous	115/35	1.00 (0.59–1.70)	0.990		
Disease status					
Recurrent vs. de novo metastatic	32/129	0.66 (0.40–1.10)	0.110		
Metastatic site					
Nonvisceral vs. visceral	47/114	1.08 (0.69–1.70)	0.738		
Liver metastasis (no vs. yes)	143/18	1.06 (0.59–1.90)	0.852		
Brain metastasis (no vs. yes)	127/34	1.05 (0.65–1.69)	0.859		
Bone metastasis (no vs. yes)	88/73	1.02 (0.69–1.52)	0.917		
PD-L1 TPS (≥50% vs. <50%)	71/90	0.89 (0.57–1.38)	0.592		
Treatment type			0.075		0.147
Dual ICI vs. chemoimmunotherapy	22/65	0.68 (0.32–1.44)	0.317	0.36 (0.12–1.05)	0.062
ICI monotherapy vs. chemoimmunotherapy	74/65	1.40 (0.90–2.18)	0.138	0.88 (0.47–1.64)	0.685
PIV (high vs. low)	79/82	1.33 (0.89–2.00)	0.165		
SII (high vs. low)	77/84	1.57 (1.05–2.36)	0.029	1.90 (1.17–3.08)	0.009
LIPI group			0.272		
Intermediate vs. poor	86/21	0.62 (0.33–1.16)	0.133		
Good vs. poor	54/21	0.60 (0.31–1.17)	0.136		
CALLY (high vs. low)	90/71	0.51 (0.32–0.80)	0.004	0.53 (0.33–0.86)	0.010

Abbreviations: HR, hazard ratio; CI, confidence interval; ECOG PS, Eastern Cooperative Oncology Group performance status; PD-L1 TPS, programmed death-ligand 1 tumor proportion score; ICI, immune checkpoint inhibitor; PIV, pan-immune–inflammation value; SII, systemic immune–inflammation index; LIPI, Lung Immune Prognostic Index; CALLY, C-reactive protein–albumin–lymphocyte index. HRs are presented with 95% CIs. Sample sizes for binary comparisons are reported in the same order as the categories listed in the Variables column. Variables with *p* < 0.10 in the univariable analysis were entered into the multivariable model.

## Data Availability

The original contributions presented in this study are included in the article. Further inquiries can be directed to the corresponding author.
